# Trans Women and Public Restrooms: The Legal Discourse and Its Violence

**DOI:** 10.3389/fsoc.2021.652777

**Published:** 2021-03-31

**Authors:** Beatriz Pagliarini Bagagli, Tyara Veriato Chaves, Mónica G. Zoppi Fontana

**Affiliations:** ^1^Laboratory PoEHMaS (Politics, Enunciation, History, Materialities, Sexuality), Institute of Language Studies, University of Campinas, Campinas, Brazil; ^2^Department of Linguistics, Institute of Language Studies, University of Campinas, Campinas, Brazil

**Keywords:** transgender rights, bathroom laws, discourse analysis, sexual violence, privacy, discrimination, security, public restrooms

## Abstract

Safe access to public restrooms is an essential need for participation in civic life, in the workplace, in educational settings, and other public spaces. This is no different for transgender people. However, access to public restrooms according to gender identity has sparked controversy to the extent that transgender people face embarrassment and even expulsion from these spaces. The lack of access of the transgender population to public restrooms has a negative impact on the physical and mental health of this population. Thus, this article aims to address the main consequences that the ban on the use of bathrooms has for the transgender population, specifically the access of transgender women to the women's restroom. We covered some legal aspects of “bathroom laws” and the main arguments in this discussion. We understand that the prohibition of access to the restroom constitutes a form of gender violence and discrimination, as we conclude that the arguments that express concerns about safety are not supported.

## Introduction

At the restroom door, the security guard came to me and asked for my documents. I replied: “why?” He said: “you know why”. I replied: “I don't know”. So I went into the bathroom with my friend and I suddenly realized that the bathroom was being evacuated. I was alone in the bathroom. He sent a cleaning woman into the bathroom and asked everyone to leave. At the time… I think that was the biggest humiliation I went through in my whole life and, believe me, I've been quite humiliated. Because he treated me as if I were a delinquent, but not just any delinquent, a highly dangerous one, who might risk those people, so dangerous that a public place needed to be evacuated^1^ (Maria Clara Spinelli^2^).

Once the door is closed, a white toilet, between 40 and 50 cm height, as if it were a perforated ceramic stool that connects our defecating body to an invisible universal cloaca (Preciado, 2018).

Surveillance, violence, humiliation, embarrassment, trauma, and suffering are everyday actions and affections in the lives of some individuals who need access to public restrooms in Brazil and throughout the world. Preciado (2018) notes that it is when architecture seems to harmlessly serve basic natural needs that a perverse and effective policy of access restriction is established, in which doors, windows, furniture, walls, partitions, exits, and entrances work as a complex apparatus at the service of *technologies of gender*^3^. Just as there is extensive research in Gender Studies regarding the complex network of constraints involving the presence of women in public spaces^4^, it is urgent to analyze the policy of transphobic spatial segregation that permeates many practices and functions, which has as one of its most violent exclusion and segregation devices in the access to public restrooms. The language in social practices and subjective relationships actively participates in these exclusion devices.

From the account of Maria Clara Spinelli, we have a sample of how discrimination operates. It is a complex apparatus that involves not only the State and its institutions, but the smallest and singular dimension—although not the less cruel—of everyone who authorizes themselves to be the “inspector of other's gender.” This discourse involves, for example, the security agent of a shopping mall, who, from misunderstanding games of glance, recognizes certain individuals as subjects, and authorizes himself to question them, demanding their documents, saying “you know why.” It is by returning to the other the evidence of historical violence that the arguments turn into a “you know why,” closing the door and locking the other inside the very own violence that victimizes them.

Language helps us realize how certain ideological processes materialize, and, in this case, we are facing a very familiar functioning. According to Pêcheux (1975/2009), it is not just about “everyone knows”—i.e., fundamental ideological evidence—, but the “you know,” which implies an enunciative game in which the subject is placed as accomplice of the violence that affects them: “you and I know why^5^.” A perverse game that finds shelter in the social relations, as all the women present agree with the scene and participate in it, leaving the bathroom. We ask ourselves: what if they stayed? What if, by staying, they showed the security guard that the only dangerous thing was his prejudiced attitude? And that the assumption of a danger and threat say much more about who acts that way and where their desire rests? Maria Clara remembers that the restroom is a public space. Are we really willing to live together?

This account, or rather, this outburst, is available on Youtube, which confirms that there is a voice, a face, a body giving life to those words, faltering in the syntax, exposing how disturbing it is to express oneself in a traumatic experience. If we consider enunciation, we can notice when the speech trembles, when the pause interrupts the word, when the nervous laughter is followed by the expression: “and, believe me, I've been humiliated a lot.” The most humiliating episode in Spinelli's life takes place at the entrance of a “Women's” restroom, as the door sign indicated. And we know that crossing that door, or rather, crossing that border, says much more about the subject's relationship with desire—by a psychoanalytic (Allouch, 2010) perspective—and the subject's relationship with a naming process, which is part *of a repeated norm* (Butler, 1993/2013, p. 161), than a biological, anatomical, or genetic data.

The bathroom is part of the exclusion operation of cities and, as Preciado (2018) points out, it is necessary to think of the historicity of the public bathroom as a bourgeois institution responsible the management of bodily waste, especially from the nineteenth century onwards, which emerges in accordance with conjugal and domestic codes crossed by the spatial division of gender, the normalization of heterosexuality^6^ and the pathologization of homosexuality: “[…] In the twentieth century, bathrooms became authentic public inspection cells, in which the adequacy of each body with the current codes of masculinity and femininity is evaluated^7^” It is as if an unwritten law authorizes people going to the bathroom to inspect the bodies of those who choose to cross the border that separates the inside and the outside (of the door and of gender).

We have a significant sample that such violence practices operate daily not only on the doors of public restrooms, but through a set of statements surrounding those places, like the speech of state deputy Douglas Garcia when he stated during a session in the Legislative Assembly of São Paulo in April 2019, that: “if by chance inside a woman's bathroom, that my sister or my mother is using, a man who feels like a woman or who may have taken off or put whatever he wants on, enters, I don't care: I'm going to beat him out of there first and then call the police” (Huffpost Brasil, 2019). The deputy also said that it was necessary to respect “the biology and values of our people.” This statement puts at stake a series of meanings that not only make invisible and deny gender identity by erasing the designation “trans-person” or “transvestite” by referring to them as “a man who feels like a woman or who may have taken off or put whatever he wants on,” as it also shows how this issue is crossed by moral arguments, since the deputy uses his supposed family responsibility (as a brother and son) to justify his conduct in face of this type of situation^8^. This is a conduct that, incidentally, also raises not only the violence of what was said—“beat him out of there first and then call the police”—, but also the violence of what was silenced: what was taken off? What was put on? There we have the cynical modesty that forbids the enunciation of the names of the genitalia as a counterpoint to the authoritarian shamelessness to openly incite violence.

As a result, São Paulo state deputy Erica Malunguinho, a trans woman, filed a lawsuit for breaking parliamentary decorum that resulted in a verbal warning against Deputy Douglas Garcia by the Legislative Assembly's Ethics Council (Huffpost Brasil, 2019).

In our theoretical course, we seek to foster possible dialogues between the field of materialist discourse analysis and feminist and gender studies. This dialogue allows us, on the one hand, to take language as the place of materialization of ideological processes (Pêcheux, 1975/2009), to question the logically stabilized universe of discursive constructions regarding events, questioning the functioning of ideology, its contradictions, and the evidence that essentialize the subjects and their effects of meanings in History. On the other hand, the political and theoretical work of Feminist and Gender Studies allows the denaturalization of the notion of identity as something pre-discursive, natural and biological, interrogating the ways in which the subjectivity of the gendered subject is historically constructed. From an analytical point of view, our view goes through several enunciative instances (legal documents, testimonies, audiovisual productions), taking into account the significant specificities. This gesture seeks to work on the events linked to gender violence in its multiplicity, showing the heterogeneity of the discursive processes, their contradictions, dominances, and resistance movements, without any instance overlapping the other.

Considering the aforementioned, this paper aims to address the controversy over the restriction of restroom use according to gender identity by the transgender population^9^, specifically by transgender women. We analyzed some legal aspects of the so-called “bathroom laws” and the main arguments in this discussion, especially those related to the allegations of risk to other women in those bathrooms. We understand that the lack of access or the prohibition of access to restrooms is a type of gender violence that negatively impacts the presence and circulation of transgender people in different social spaces, resulting in segregation and ghettoization of this population. At the same time, we analyze the process that constitute what support such prohibition policies, processes that reinforce historically dominant meanings about masculinity and femininity, and that build the image of a subject-other, upon which meanings of violence (particularly sexual violence) and animality are projected.

## Bathroom Law

Safe access to public restrooms is a right and a necessity for participation in civic life, in the workplace, in educational settings and other public spaces. However, many transgender people are afraid to go to bathrooms, as they are exposed to embarrassment (and violence) and may even be prevented from accessing them. This stems from discriminatory practices already socially established and not legally regulated, given the absence of clearer and/or effective laws or legal provisions that protect the rights of transgender people to access these spaces without embarrassment or hostility. Therefore, the right to access bathrooms is fundamental to the fight for equality in the transgender community, which is reveal by the many legal cases that dealing with protection against discrimination that refer to this issue (Elkind, 2006, p. 922).

The legal debates about the right to use restrooms by transgender people in the United States add to the set of studies known as the “bathroom law” or “bathroom bill,” which adds legal provisions and analyses ranging from the right to work to the dismantling of the racial segregation experienced in that country^10^ (Rios and Resadori, 2015, p. 204). Levi and Redman (2010, p. 133) go so far as to say that “bathroom inequality is one of the greatest barriers to full integration of transgender people in American life.” Rios and Resadori (2015, p. 204) argue that the accumulation of the American legal debate on “bathroom laws” provides valuable arguments for improving this discussion in the Brazilian context.

Trans-exclusionary bathroom laws (or bills)^11^ end up giving new meaning to these equipment by targeting its use exclusively to cisgender people, segregating the transgender population as a result. These discriminatory initiatives reverse the burden of crime, by penalizing trans people who assert their right to use social facilities, instead of penalizing the very discriminatory parliamentary practices that intend to legislate for the exclusion and invisibility of this population. They are usually based not only on a definition of sex as a set of physical characteristics seen as *immutable*, but also on the legal assignment of sex registered in a person's first birth record. For example, a bill presented in South Carolina—United States—^12^ understood that the “*original birth certificate may be relied upon as definitive evidence of an individual's sex.”* The emphasis on the *original* birth certificate as *definitive* evidence is not accidental, as transgender people, including minors, may have later rectified the assignment of sex in their official documents^13^. The proposed wording therefore implies that even transgender people who have already managed to rectify their documents could not, in theory, use the bathrooms in accordance with their current official documentation, which denies the right to recognition of civil identity and legal status of transgender people.

On the other hand, it is noteworthy that no bathroom law has so far been able to explain how and by whom a person's gender would be effectively verified [Movement Advancement Project (MAP), 2016, p. 4] in everyday contexts of using restrooms. This is especially salient for trans-exclusionary laws that are based on a notion of sex as physical or chromosomal anatomy. In this regard, laws that forbid the use of restrooms due to gender identity are impossible to enforce, unless the government is willing to engage in invasive policing of the use of restrooms by its citizens [Movement Advancement Project (MAP), 2016, p. 9] or endorse people to informally “watch” each other, promoting a social suspicion environment. Those surveillance practices necessary for the application of trans-exclusionary bathroom laws are based on the idea that it is evident the determination of someone's access to restrooms through bodily characteristics (Beauchamp, 2019, p. 106), and that transgender people practice “gender fraud.”

The term “trans-exclusionary” has often been used to specify radical feminist currents that advocate the exclusion of trans women from feminism, which includes the acronym *TERF* (trans-exclusionary radical feminist) (Bagagli, 2019). This exclusion is based on the basic premise that the fight for rights of transgender people is antagonistic with the rights of cisgender women. In addition to the naming of this feminist current, the use of the expression “trans-exclusionary” is capable of designating an extensive set of transphobic practices that defend exclusion or effectively exclude transgender people from different spaces, which includes, in the scope of the analysis of this work, the exclusion of trans women from the women's bathroom. In this sense, we understand that the exclusion of transgender people due to their gender identities is a form of manifestation of transphobia and/or cissexism. It is not by chance that several trans-exclusionary radical feminists advocate positions favorable to the exclusion of trans women from women's bathrooms. According to Jones and Slater (2020, p. 835), over the last decade, hostility directed toward trans people from some factions within feminism has monopolized public discourse around the movement and the access to the toilet has thus become a symbol overloaded with significance.

Some authors consider a distinction, although slight, between transphobia and cissexism. While transphobia, for Serano (2016), implies fear or aversion, broadly, to all identities, expressions, appearances, and behaviors related to gender that deviate from social norms, cissexism is based, more specifically, on the belief that the gender identities of transgender people are inferior or less authentic than the identities of cisgender people. Kaas (2012), on the other hand, understands that transphobia refers more usually to the most obvious and ostentatious examples of discrimination and violence against transgender people, while cissexism relates to discourses and practices that invalidate transgender identities in a subtler or veiled way.

Free access to the bathroom without the fear of being embarrassed or expelled due to one's gender identity can be described as a form of cisgender privilege. The cisgender (or cissexual) privilege is thought by Serano (2016) through the action of a “double standard that promotes the idea that transsexual genders are distinct from, and less legitimate than, cissexual genders.” The act of gendering, defined by Serano as the process of distinguishing between females and males in which “we actively and compulsively assign genders to all people based on usually just a few visual and audio cues” has a central role in establishing the tacit rules of the use of the bathroom according to gender. The condition of invulnerability to misgendering^14^ is, in general, a cisgender privilege. Transgender people, particularly those who “pass” as cisgender, can enjoy conditional cisgender privilege, because although they may have their genders legitimately recognized, this can be threatened from the moment their transgender condition is revealed or addressed. The need to “pass” as cisgender, in the context of using the bathroom, aims to circumvent the stigmas, both visible and hidden, associated with gender non-conformity and is carried out through a continuous act in everyday life (Kessler and McKenna, 2000, p. 17).

Preciado (2018) reminds us of the existence of this kind of unwritten law that allows everyone to publicly control femininity, initially through looking and, when in doubt, through the speaking: “hey, hey, you're at the wrong door,” “the men's bathroom is over there,” among other more or less cynical statements that insist on putting the gender “inside the box.” This process of interpellation crossed by the look and the power of the word concerns a complex network that involves those who feel entitled to speak—why would they feel in agreement with their gender?—and approach the other, not anywhere, but exactly where the choice is binary: male or female. In the case of Deputy Douglas' speech, he not only poses himself as a “law enforcement” of the other's gender, but at the same time his argument is justified “by the women” taken care by him: sister and mother, women figures that allegedly need male protection.

## Consequences of Hostility Against Transgender People and the Legal Aspects of Using Restrooms

We assume that the laws, measures, and positions that support the prohibition of transgender people accessing bathrooms according to their gender identities are expressions of hostility and discrimination^15^ against this group. As Machado (n.d.) points out, hostility toward transgender people, particularly regarding the use of public bathrooms, inhibits not only the use of the bathroom itself, but also the presence and circulation of trans people in several other spaces, including schools, work, and leisure areas. The journalist also points out that failing to go to the bathroom when necessary is one of the risk factors for urinary tract infection, which can affect the bladder, ureters, urethra, and kidneys. The lack of safe access to bathrooms by transgender people is also associated with mental health problems, conditions related to stress, and increased levels of suicidal thoughts and behavior (Herman, 2013).

The binary conception of gender that underlies the spaces segregated by gender ignores or marginalizes those people who do not fit the norms of gender expression, whether they are transgender or cisgender. These people can be seen as being in a “wrong” bathroom, whether male or female. Kogan (2008) understands that the very binary division of bathrooms between male and female impacts on the way bodies and gender identities are interpreted, as this division is seen as the unquestionable evidence that human bodies can only be male or female. Thus, bodies that do not easily fit into this binary classification are considered unacceptable and can be ordered to leave those places. Black transgender people showed higher rates of exclusion and embarrassment in bathrooms than white transgender people (Herman, 2013), which indicates that the fight for the right to access the public bathrooms must also consider race and class (Patel, 2017).

Bathrooms segregated by gender implicitly shows that there are only two possible forms of gender expression and, therefore, restrict public acceptance of transgender individuals who defy social norms (Rudin et al., 2014, p. 724). On the other hand, the heightened and recent debate on the use of restrooms by transgender people is also seen with surprise, considering that transgender people have already used public bathrooms for countless years without other people noticing them. But the question here is not only related to the historical existence of trans individuals in society, but to the fact that in the current conditions of production, the conditions of existence, permanence, and circulation of such individuals in the public space go beyond the everyday conversations, and public and private institutions debates. When we turn to the current political scenario in Brazil, we know that the discussion about sexuality and gender goes beyond the walls of epistemological productions and disputes over identifications that have marked the theoretical and activist field in gender and Queer studies (cf. França et al., 2019). Such discussion also concerns a reactionary wave that marks the current political debate in Brazil, its electoral platforms and the evangelical groups in the national congress, which have a position regarding what “already existed without people realizing it.” To talk about it means that processes that cross language and history, such as nominations, designations, activism and theoretical productions, videos, poetics and aesthetics, among others, disturb the meanings already established on the issue.

For the United States Department of Labor Occupational Safety Health Administration (2015, p. 1), restricting transgender employees to only use bathrooms that are not consistent with their gender identity, or segregate them from other workers, requiring the use of gender-neutral bathrooms or other specific bathrooms, isolate these employees, and may make them fear for their physical safety. As a result, the agency recommends that all workers, including transgender workers, should be able to access bathrooms that match their gender identities. However, in the United States, measures that protect access to the bathroom by transgender people vary by state, and there is no federal law associated [Movement Advancement Project (MAP), 2016]. The controversies generated by the use of restrooms by transgender people have unfortunately been used for some employers to fire transgender employees (particularly those who start their gender transitions after being employed) or to avoid hiring them, aggravating discrimination, and social exclusion.

A survey (James et al., 2016) carried out with 28,000 transgender or diverse gender people, with 18 years old or more in the United States in 2015, showed the following situations experienced up to 1 year before the research: 48% *sometimes* avoided and 11% *always* avoided using the bathroom, totaling 59%; 32% limited their drinking habits to avoid using the bathroom; 24% had their presence in a particular bathroom questioned or challenged; 12% were verbally harassed, physically attacked, or sexually abused when accessing or using a bathroom; 9% had access to the bathroom effectively denied, with undocumented residents (23%), and interviewees working in the clandestine economy (20%) (such as sex work, drug sales, and other currently criminalized jobs) being twice more likely to be denied access to restrooms than the general sample; and 8% reported having a urinary tract infection, kidney infection or other kidney-related problem as a result of avoiding using the bathroom.

Rios and Resadori (2015, p. 200) cite judicial cases (until 2014; Brasil, 2014) of Brazilian trans or transvestite women who were prevented from using public female restrooms and had their indemnity lawsuits denied by the State due to the understanding that they would not have suffered discrimination, embarrassment, psychological, or moral harassment. This understanding, however, is based on the premise that transgender and transvestite women are “in fact” men, and therefore could not denounce the impediment to accessing the women's bathroom as discrimination. On the other hand, transsexual and transvestite women have also won victories in their claims for moral damages due to the restriction of using women's bathroom, showing that these decisions still diverge in the Brazilian courts.

The Brazilian Supreme Court (STF) recognized in 2014 that the use of bathroom by transgender people is a general repercussion thesis resulting from the Extraordinary Appeal (*Recurso Extraordinário*—RE) (845779), which, in turn, seeks to reform the Court of Justice of Santa Catarina (2012) decision that had dismissed an indemnity lawsuit for moral damages to a transgender woman that was forbidden to enter a female bathroom in a shopping center and who, shaken by what happened, ended up urinating in her own clothes, in front of everyone there (Rios and Resadori, 2015, p. 203). The Court of Justice of Santa Catarina (TJ-SC) understood that there was no moral damage, but “mere dissatisfaction” (Notícias STF, 2015). The legal question, therefore, is to determine whether the requirement that a transgender person use designated to the gender they do not identify with is an offensive conduct against the dignity of the human person and personality rights, and therefore indemnifiable as moral injury (Notícias STF, 2014). Rios and Resadori (2015, p. 210) argue that simply ignoring transsexuality in a space as meaningful and vital as public bathrooms implies disregarding or excluding transgender people due to their gender identities and also hurting the heart of the constitutional protection of human dignity.

However, the lawsuit has not been completed so far, as it was interrupted in 2015 by a request for a review from Minister Luiz Fux (Notícias STF, 2015). At least 778 similar cases, currently suspended, would be concluded with the decision of the RE (Notícias STF, 2015). One of the justifications for this request for review and this interruption is that the matter would generate, according to the minister, a “reasonable moral disagreement” so that “social opinion” should be considered on the topic. Minister Luís Roberto Barroso had proposed the following thesis for general repercussion: “transsexuals have the right to be socially treated according to their gender identity, including the use of public bathrooms.” The opinion of the Attorney General's Office had also concluded that “it is not possible for a person to be treated socially as if they belong to a different sex from which they identify with and present themselves publicly, as sexual identity finds protection in personality rights and dignity of the human person.”

Carvalho Filho (2015) is surprised before Minister Fux's argument, because, according to the author, there is no glimpse of reason in an eventual moral disagreement in view of the inexistence of a plurality of constitutionally legitimate options in the case under analysis. The author points out that reasonable moral disagreements are constituted by the “lack of consensus on controversial topics whose antagonistic solutions are constructed as rational products,” thus involving “diverse positions that coexist within society,” but which are equally legitimate constitutionally.

The absence of a determination by the Supreme Court of Brazil on this issue allows for municipal laws to be passed aimed at forbidding the use of restrooms by transgender people according to their gender identities, such as Law No. 7,520 of Campina Grande (Paraíba) signed on May 25, 2020 by Mayor Romero Rodrigues (Campina Grande, 2020), which, by prohibiting the “interference of ‘gender ideology^16^, in public and private elementary schools,” had determined that the use of bathroom, locker rooms, and other spaces in schools should “continue to be used according to the biological sex of each individual, with any interference of the so-called ‘gender identity' being prohibited,” and establishing fines to the School Manager or the school owner (if private) if the law was not met. However, a preliminary decision granted by the Justice in a public civil action filed by the Human Rights Nucleus of the Public Defender of the State of Paraíba on June 10, 2020 annulled the application of fines to schools in Campina Grande that allow the use of bathrooms in accordance with the gender identity of young transgender or diverse gender people and also determined that students can use bathrooms in accordance with their gender identities (G1 PB GLOBO, 2020). Another similar municipal law, in Sorocaba (São Paulo), was considered unconstitutional by the São Paulo Court of Justice (Viapiana, 2019).

Despite the absence of a comprehensive and nationwide resolution, it is noteworthy that Resolution No. 12 of the National Council Against Discrimination and for the Rights of Lesbians, Gays, Transvestites, and Transsexuals of January 16, 2015 (CNCD, 2015), when establishing the parameters to guarantee the conditions of access and permanence of transvestite and transsexual people in educational systems and institutions, decided that the use of bathrooms, locker rooms, and other spaces segregated by gender must be in accordance with each person's gender identity. Within the specific scope of the Federal Public Ministry (MPU), Ordinance No. 7 of March 1, 2018 of Attorney General's Office of Brazil establishes that the use of bathrooms, locker rooms, and other spaces segregated by gender is guaranteed according to each individual's identity. This ordinance includes service users, members, employees, interns, and outsourced workers under the MPU (Brasil, 2018).

On October 14, 2020, the Attorney General's Office (AGU) sent an appeal (embargoes of declaration) to the Supreme Court of Brazil to clarify points of the trial that framed homophobia and transphobia within the racism law. The AGU seeks to find out to what extent the criminalization of prejudice against LGBT people affects religious aspects. The Supreme Court's decision had already determined that “freedom was ensured so that religious leaders can argue in their cults that homoaffective conduct is not in accordance with their beliefs, as long as such manifestations do not constitute hate speech, thus understood the externalizations that incite discrimination, hostility or violence against people because of their sexual orientation or gender identity” (Folha de S. Paulo, 2020). According to Amparo (Folha de S. Paulo, 2020), the federal government intends to expand exceptions to the criminalization of homophobia and transphobia. One of the points that the AGU demands explanations for refers precisely to “the control of access to certain places open to the public (such as bathrooms, locker rooms, penitentiary establishments, and public transportation wagons)” and understands that “the control of access to certain places open to the public based on physiobiological aspects should not be characterized as an act of racism when the restriction of entry has been established in favor of protecting the privacy of vulnerable groups,” assuming, therefore, that “the access to public spaces can be organized based on the physiobiological criterion of gender, and not on the social identity of the user” (Advocacia-Geral da União, 2020, p. 36–37).

In the USA, most trans-exclusionary bathroom bills are not approved, with the notable exception, for example, of the House Bill 2 (HB2) in 2016 in North Carolina, which determined that individuals should use the bathroom that corresponded to the sex originally assigned on their birth certificates in this state. This law was a response to a regulation (*ordinance*) in the city of Charlotte that had established anti-discrimination measures that included using the bathroom according to gender identity. In 2017, HB2 was revoked by House Bill 142, which, however, also vetoes local governments to approve anti-discrimination measures for the use of bathrooms until December 1, 2020 (Barnett et al., 2018, p. 233). The Trump administration recently caused the U.S. Department of Justice to rescind the Obama administration's position that established that non-discrimination laws require schools to allow transgender students to use bathrooms that match their gender identity (Peter et al., 2017).

Lopes (2017) and Wilson (2016) understand that the fear spread around the use of bathrooms by transgender people was a widely popular strategy used by conservatives to stop measures aimed at combating discrimination against LGBT people in the United States. It is worth remembering that access to bathroom is part of the protections against discrimination, but anti-discrimination measures or laws address more issues than just this one [Movement Advancement Project (MAP), 2016, p. 2]. For Wilson (2016, p. 1386) “the bathroom narrative has emerged [since 2008] as the main rhetorical weapon against protecting LGBT people from discrimination in public places [in the United States].”

When we look back at the arguments that support trans-exclusionary legal measures, we face many evidence based on a pre-discursive conception of sex, in Butler's (1990/2017) terms, the idea that sex is a gross matter, unquestionable, linked to nature, therefore excluded from the social context, where gender would fit in. At the same time, such arguments are produced within a contradiction that arises from the imaginary around masculinity and femininity that constitute the “bathroom narratives”: the idea that women are defenseless and men are aggressive by nature; then, we face a set of attributes that makes the social and the nature contexts not that far from each other. Thus, perceiving the way sex is part of the argumentative plot of such measures is one of the ways of realizing two mechanisms that work together, one supporting the other: (1) a transphobic ideological process based on the very denial of ideology to make nature as the only truth of things and (2) a process of sedimentation of cisnormativity by dichotomizing and naturalizing what would be biologically feminine vs. what would be biologically masculine.

## Trans-Exclusionary Positions on the Use of Restrooms

We can identify two aspects of trans-exclusionary positions in relation to the use of women's bathrooms: (1) the defense of laws or measures that effectively aim to prohibit the access of trans people to restrooms according to their gender identities (trans-exclusionary bathroom laws); and (2) opposition to laws or measures that explicitly guarantee access for transgender people to bathrooms according to their gender identities without constraint or discrimination (trans-inclusive bathroom laws). Thus, we infer that positions that defend trans-exclusionary laws necessarily oppose to trans-inclusive measures; however, not all positions that oppose the establishment of trans-inclusive measures necessarily advocate explicitly trans-exclusionary measures.

We will see next how dominant meanings regarding masculinity and femininity support what we call here trans-exclusionary arguments regarding access to public bathrooms. The violence argument is a constant in this discussion, although statistics show that there is no concrete data to prove that trans people are a threat or participate on acts of violence against users of women's bathroom. So, why do such arguments continue to support argumentative and, therefore, discursive processes that segregate and exclude transsexual women not only in public bathrooms, but in the many social practices and spaces? We know that Brazil has a history of violence crossing the relationship between women and the public space, and our political and theoretical position about it does not deny or erase such historicity. On the contrary, it allows us to think about how the violence acts at the intersection between historical determinations involving gender, sexuality, race, and class around the condition of abjection^17^.

Some proposals of apparent consensus aim at the creation of a third bathroom, which would then be destined for transgender people at the expense of the use of female (in the case of trans women) and male (in the case of trans men) bathrooms. However, despite the possible use of these bathrooms by transgender people whose gender identities do not fit into gender binarism and as an intervention that proposes to legitimately question the binary division of restrooms (without assuming that transgender people should be forced to use only neutral bathrooms) this proposal is potentially problematic. According to Elkind (2006, p. 927):

The proposal for a third category of gender neutral facilities is not the solution. The proper means of attaining transgender equality is not to segregate the group into an extraneous “other” category, but to treat transgender individuals as the majority is treated and to permit each person bathroom access based on his or her gender identity. Gender neutral bathroom access is both cost prohibitive and ignores the underlying problem faced by transgender individuals with respect to bathroom access. Individuals should be considered as members of the gender group with which they identify and not as an abnormal “other” denied recognition among existing societal groups. Creating a third group of gender neutral bathrooms for transsexuals only bolsters the assertion that such individuals do not “fit in.”

One of the arguments for transgender people to not use the bathrooms according to their gender identities is that it could generate some kind of embarrassment for other people (presumably cisgender) using the space (Rios and Resadori, 2015), or even that security, specifically in the case of women's bathrooms, could be impaired^18^. Whether for security or privacy^19^, the underlying message that emerges in these speeches is that trans people are disregarded on the one hand in relation to their affections (why would they not feel embarrassed?), and, on the other hand, perversely stigmatized, because they are considered sexually threatening^20^ (Levi and Redman, 2010, p. 144). The legislative position that conceives transgender bodies as threats requires complicity with pervasive practices of surveillance in bathrooms, which spread, at the same time, the idea of cisgenerity as the standard of normal bodies, easily interpretable and inherently compatible with the use of bathrooms without constraint (Beauchamp, 2019, p. 106). From this perspective, the access of transsexual and transvestite women to women's bathrooms would mean the supposed permission for “men” to also access these spaces (assuming, with this discourse, that transsexual and transvestite women are simply men because they share some biological characteristics) and eventually abuse other women in bathrooms.

It is relevant to point out that, for positions that do not conceive transgender women as men, the claims that measures aimed at guaranteeing trans women access to women's bathrooms would allow men to access women's bathrooms make no sense. In a consensus statement [National Task Force to End Sexual and Domestic Violence against Women (NTF), 2018] against laws that prohibit the use of bathrooms due to gender identity signed by more than 300 U.S. organizations that fight against sexual and domestic violence, we read that:

Nondiscrimination laws do not allow men to go into women's restrooms—period. The claim that allowing transgender people to use the facilities that match the gender they live every day allows men into women's bathrooms or women into men's is based either on a flawed understanding of what it means to be transgender or a misrepresentation of the law.

Also according to the declaration, the idea that protection for transgender people (including using the bathroom without constraint due to gender identity) harms the privacy and security of other users is a myth. Several critics point out that there is no evidence that non-discrimination policies or that explicitly allow transgender people to use restrooms according to their gender identities have led to an increase in the number of sexual harassment cases in bathrooms and women's locker rooms anywhere in the world (Doran, 2016; Hasenbush et al., 2019). States (19) and cities (more than 200) in the US that have passed laws against discrimination against LGBT people show that such measures have not caused any increase in incidences of crime in bathrooms (Maza and Brinker, 2014). This is not surprising, given that the approval of protections against discrimination has no impact on existing laws that criminalize violent behavior in bathrooms. In the absence of real incidents to base trans-exclusionary bathroom policies, anti-trans groups fabricate horror stories about trans-inclusive bathroom policies (Maza, 2014).

Security and privacy in the use of public restrooms are certainly important for everyone—including transgender people. Arguments that unilaterally conceive the access of transgender people to restrooms according to their gender identities as a risk factor for the safety of other people assume, even implicitly, that the transgender population does not deserve to be protected under the same standards as the cisgender population. This is particularly alarming, given that research shows precisely that young transgender people are exposed to much higher rates of violence in US schools' restrooms (*middle and high school*) than young cisgenders (Murchison et al., 2019).

The safety in the use of restrooms can only be effectively compromised through attacks by abusers, so it is misleading to simply assume that transgender people, especially transgender women, commit these crimes or are essentially more predisposed to commit such crimes only because they access women's bathrooms or for not having explicitly denied their access to women's bathrooms by law or regulation. It is worth remembering that sexual harassment and rape are already considered crimes, so it does not seem reasonable to create new laws to curb crimes that have already been typified. Violence cases can happen and/or happen in restrooms regardless of the approval of trans-inclusive bathroom measures or laws. People should be held responsible for any crimes in bathroom spaces regardless of gender identity, whether transgender or cisgender, and which people (or groups of people) have access to a particular bathroom. Among bathroom attack cases, only a small number of cases actually involved transgenders, people who^21^ falsely claimed to be transgender or perpetrators who tried to disguise themselves as a member of the opposite sex to gain access to the bathroom (Barnett et al., 2018, p. 235). Thus, the idea that it is necessary for individuals to use bathrooms according to the gender assigned to them at birth to ensure safety in these spaces is inconsistent and disproportionate. In this sense, Davis (2018, p. 206–207) makes the following questions:

The assumption that sex-segregated public bathrooms protect women from physical assault is flawed in two ways. First, sex-segregated restrooms only serve as a barrier to physical assault if one's attacker is of the opposite sex. Secondly, if someone is already willing to break laws to commit criminal assault, it is likely that the person will break another law to enter a women's restroom with little or no hesitation. Public restroom sex-segregation is not the best, or even a rational, way to address the very real and important matter of anti-female violence. Even worse, the misconception of women's restrooms as places of refuge may lull many women into a false and dangerous sense of personal safety when they enter those rooms.

Despite the recent spread of the “bathroom predator” (Schilt and Westbrook, 2015; Fitzgerald, 2016) in the social imaginary and its impact on bathroom laws, it is noteworthy that most US citizens are opposed to measures that would effectively force transgender people to use a bathroom in disagreement with their gender identities (Wilson, 2016, p. 1388). This discrepancy does not seem to us to be fortuitous, since it indicates the presence of an ambivalence in the speeches that defend the prohibition of the use of the feminine bathroom by transgender women or that conceive the access of transgender women to the feminine bathrooms as a risk factor for security.

People who report some kind of fear regarding the access of transgender women to the women's bathroom may simultaneously recognize that it would be wrong, on the other hand, to force trans women to attend the men's bathroom. Many still admit that it would not be transgender women who would actually commit sexual crimes in restrooms, rejecting the idea that this particular group (transgender women) would directly represent a risk factor for safety in the bathrooms, but rather the men who would falsely claim being transgender women, that is, men who would somehow inadvertently benefit from anti-discrimination measures to commit such crimes. In this way, abusers would supposedly have facilitated access to victims by measures that guarantee access for trans women to women's bathrooms and/or the sheer absence of measures that explicitly prohibit access for trans women to women's bathrooms.

In a statement by the Massachusetts Family Institute (MFI) (Levi and Redman, 2010, p. 142) against anti-discrimination laws on bathroom use, we read that “there is no way to distinguish between someone suffering from ‘Gender Identity Disorder' and a sexual predator looking to exploit this law.” If we assume that there would be no way to “distinguish” transgender women from sexual predators, we are very likely to conclude that it is necessary to prohibit transgender women from accessing women's bathrooms because of the maintenance of security. However, if we really wanted to apply this argument without a cisnormative bias, we would have to the same extent recognize that we could not also distinguish, in an absolutely unequivocal way, cisgender women from sexual predators. The fact that we cannot guarantee with absolute unmistakability who may or may not be a potential “sexual predator” seems to have a burden only for transgender women. In this sense, under the operation of the most elementary evidence in relation to gender, we are not equally likely to conclude that female cisgender women should no longer share the use of the female restroom due to the possibility of female cisgender women committing crimes or being “sexual predators” in the bathrooms in the same way as we do with transgender women. These cisnormative biases, therefore, should not go unnoticed without critical analysis when discussing these arguments. Jones (2015) exposes this bias as follows:

In most cases, we understand that allowing any group of people into a given place means that some small fraction of them might commit crimes, and we accept that the benefits of their being able to access that place outweigh the potential risks. Cis women have assaulted cis women in restrooms, yet nobody takes this as a reason to ban all cis women from women's restrooms. Imposing that kind of inconvenience on all cis women is obviously unacceptable, but imposing it on trans women is totally okay for some reason. (The reason is transphobia.)

If we assume that the absence of laws or measures that forbid access for trans women to women's restrooms is in any way an incentive for sexual predators to pretend to be trans women to access their victims in those places, then we should face such cases on a daily basis, considering that most countries or states around the world do not actually have laws or measures that explicitly prohibit transgender women from accessing women's restrooms, nor do they have measures that establish ways to effectively bar trans women from accessing restrooms or checking whether the women who are accessing those bathrooms had the female gender signed on their first birth certificate. However, this does not appear to be the case, given that there is no evidence that the absence of laws or measures that prohibit the use of the women's bathroom by trans women may in fact represent a risk factor for safety in these spaces, nor that approval of measures against discrimination against transgender people in bathrooms has some impact on the chance of people violating criminal laws regarding rape and sexual harassment. Jones (2015) makes the following question: whether under trans-inclusive laws sexual predators can pretend to be transgender women to access the women's bathroom, which would prevent them, under trans-exclusionary laws, from pretending to be transgender men to access the same women's bathrooms? According to Wilson (2016, p. 1401) the connection between the supposed implications of safety in restrooms and the guarantee of access for trans women to women's bathrooms rests in a “cascade of factual assumptions” about “*situational and preferential sex offenders”* that could attack victims in facilities segregated by sex.

In a statement (Arter, 2015) of a campaign against a Texas *ordinance* that would allow the use of restrooms according to gender identity, we read that:

[This] Bathroom Ordinance would force businesses and public establishments to allow troubled men, or men who want to start trouble, to use women's public bathrooms, locker rooms and shower facilities. This endangers women and girls and places them in harm's way. There are 8345 registered and convicted sexual predators in Harris County. This just scratches the surface of this dangerous problem. These men could use this ordinance as a legal shield to threaten our mothers, wives and daughters.

Assuming that trans-inclusive bathroom laws allow “condemned sexual predators” to access women's bathrooms, this discourse produces a series of equivalence substitutions between the following elements: transgender women > men > problematic men > condemned sexual predators. The access of “condemned sexual predators” to women's bathrooms is a possibility condition for them to be able to commit sexual crimes against women in these spaces, which seems logical to conclude that it is necessary to reject trans-inclusive bathroom laws to curb attacks in women's bathrooms. Furthermore, this statement takes on the face of a masculine law, unable to conceive women simply as “women,” and not already interpreted from the social roles that supposedly link them to a man: as mother, wives, and daughters.

We propose to look at the implicit thought that transgender women are men from a pre-built idea (Pêcheux, 1975/2009, p. 159), which is understood as a content already produced, something that “everyone knows,” as well as what “everyone in a given situation can be and understand, under the evidence ‘of the situational context.”' The successive substitutions that associate transgender women with a condemned sexual predator and the consequent production of cause and consequence effects (the accessibility of transgender women to women's bathrooms *causes* the vulnerability of cisgender women allegedly exposed to attacks by sexual predators in women's bathrooms) can be understood from the notion of a transversal discourse. For Pêcheux (1975/2009, p. 152), the transversal discourse functions as a sequence that perpendicularly crosses another sequence that contains replaceable elements (in our case, transgender *women* vs. *condemned sexual predators*). This transversal discourse produces the evidence that certain biological characteristics shared between transgender women and condemned sexual predators (notably the presence of a penis and other possible physical attributes associated with the male sex) expresses a necessary condition for the practice of sexual crimes in bathrooms as if it were necessary to have these biological characteristics^22^ to practice such crimes in women's bathrooms. This is able to explain, on the other hand, the absence of similar concerns regarding the presence of transgender men, whether in men's or women's bathrooms, as well as the very assumption that cisgender women pose no threat to themselves. According to Schilt and Westbrook (2015, p. 30):

In contrast, transgender men—assumed by critics to be “really women” because they do not possess a “natural” penis—are relatively invisible in these debates. Transgender men are mentioned directly by opponents only once in all of the articles we analyzed. (…) Transgender men are never referenced as potential sexual threat to women, men, or children. Instead, they are put into a category that sociologist Mimi Schippers labels “pariah femininities.” They are not dangerous to cisgender women and children, but they also do not warrant protection and rights because they fall outside of gender and sexual normativity.

A statement against the Department of Justice under the Obama administration (which argued that HB2 violated federal law) says that “apparently, the Department believes that these obvious social costs are outweighed by the policy's purported psychological benefits to persons of conflicted gender identity” (Kogan, 2017, p. 1231). The statement assumes that the use of restrooms by trans people according to their gender identities implies an “obvious social cost” and implies that the supposed psychological benefits for trans individuals would not outweigh “obvious social costs” (in this position, risks regarding security or privacy violation) of a trans-inclusive policy. Transgender gender identities are qualified as “conflicting.”

Assuming that the “costs” that transgender women would suffer are preferable to those that cisgender women would suffer, we can consider the functioning of a valuation scale in which the protection of the dignity of cisgender women is ahead of transgender women. This cost-benefit calculation projects onto trans people an idea of second-class citizens (Beauchamp, 2019, p. 86), as well as the intrinsic vulnerability of cisgender women. When we also consider race, class and/or social situation (see James et al., 2016, p. 225), we understand that black and/or non-white transgender women, in a situation of social vulnerability and/or poverty, and also when those women do not “pass” as cisgender, they are the base of the discrimination scale. According to Beauchamp (2019, p. 98):

Legislative and public discourse on the transgender threat to other gendered bathroom users draws on familiar viewing practices that simultaneously claim bodies as objective, apolitical data points (we can easily know which bodies are women's bodies) and reiterate a decidedly social and political meaning given to different types of bodies (women's bodies are vulnerable and need special protection). Certain women's and children's bodies will more readily signal vulnerability, a point that public bathrooms themselves underscore, since the history of bathroom segregation rests largely on the protection of white women.

In this direction, another relevant aspect to the scale concerns the family and/or personal connection of a woman with a man. This is justified because of the roles attributed to women as *mothers, daughters, sisters, and wives* in speeches of men who believe they need to protect them from the supposed dangers resulting from trans-inclusive bathroom laws. In the hegemonic imaginary, men are conceived as potential protectors of vulnerable people with whom they have personal ties; and a potential source of sexual threat to other people, notably other women (Schilt and Westbrook, 2015, p. 31). According to Blumell et al. (2019, p. 383) the protection of cisgender women in the context of using the women's bathroom is seen as dependent on certain conditions, including:

(a) The defender has a personal relationship with the woman (wife or daughter), which situates her welfare within her importance to an individual man; (b) the threatening man is seen as non-normative, deviant, and uncontrollable, in perpetuation of the stranger danger rape myth (Weiss, 2009); and (c) the woman being protected is cisgender, monogamous, and heterosexual, revealing an implicit assumption that “good” women who have men to protect them will not be raped. Transwomen, by contrast, were not seen as needing protection because of their deviance and presumably masculine strength.

By basing trans-exclusionary positions on individual references that guide the family and/or personal ties, we observe a universalization effect from this male point of view regarding the ideas of protection, vulnerability, and danger. That is, successive precepts and notions from one individual evolve (which, in this case, coincides with the male position that maintains concrete ties with a specific daughter, wife, mother, or sister) to the production of a universal subject, operating an erasure of the preceding concrete situation, which would then start to think through concepts and abstractions (capable, therefore, of creating laws). This operation is named precisely by Pêcheux (1975/2009, p. 117) as *the empirical-subjectivist continuist myth*. For the author, this myth is based on the identification process: “if I were where you/he/x are, I would see and think what you/he/x see and think” (Pêcheux, 1975/2009, p. 118). In this case, we move from the need to protect the daughters, wives, mothers or sisters of an individual subject to the need to protect the daughters, wives, mothers or sisters of other men to any and all women. Under this specular game, there is a tension that cannot be resolved, since it is to be assumed that the same man who protects women with whom he has family ties represents a potential danger for other women with whom he does not have those ties. This process culminates in the defense of trans-exclusionary bathroom laws, under the supposed evidence that each and every woman is vulnerable to the attacks of each and every man and needs to be protected (sustained perception under the equivocal universalization of the expectation that every woman has a family or personal relationship with a man, or that every woman *should have* a relationship with a man to ensure her protection).

Another argument used to support trans-exclusionary positions concerns the number of people:

Are we to risk the safety of millions of women and children in public restrooms because an extremely small number of people are experiencing a mismatch between their psychology and their biology? Good public policy does not risk the physical safety of women and children because an extreme few have a preference for a different bathroom. (Turek, 2016).

Thus, the claim that there are an infinitely larger number of cisgender people corroborates the position that the supposed “costs” regarding the security of cisgender women would be, to the same extent, much greater than the “costs” regarding the security of transgender women. However, the considerations, for example, of the Federal Public Ministry of Brazil (Ministério Público Federal, 2015) provide universal constitutional guarantees, that is, arguments based on the quality of the person and not on the number of people. We observe that by understanding that gender identity is essential for the dignity and humanity of each and every person and should not be a reason for discrimination or abuse, in order to conclude that preventing the use of restrooms is the same as denying gender identity, thus violating the dignity of a transgender person.

## Conclusion

This thing, in particular, of using the women's bathroom, is a very delicate thing for me. Life has made me strong, but at the same I have my traumas, I have my ways to do things, I am full of… […] You, woman, who is watching this right now, put yourself in my shoes, as if you were a transvestite. Can you imagine how it is to go into a women's restroom and get kicked out? I have already been taken out of a toilet with my panties down, thrown out like an animal. I called the police, nothing happened, the police didn't even go. […] (Luísa Marilac^23^)^24^

The band Filarmônica de Pasárgada released a music video in 2014 for the song “Fiu”^25^, a Brazilian funk that takes place in a shed that looks more like a butcher shop located in a train station, in which people in bloodstained clothes dance and cut several pieces of meat. At a given moment in the video, the central door opens and the cartoonist Laerte Coutinho, a trans woman, appears and steps slowly across the room, decorating herself with necklaces, pinning her long hair, dancing, even though sometimes the music stops and everyone looks at her, examining her body and her presence with strangeness, or when retouching her makeup, a jet of blood splashes over her face. At this moment, Laerte cleans herself and looks at the bathroom door on which a female pictogram is printed, but under the symbol where the word “female” is supposed to be written, the first syllable “fe” is cut, so that it appears written just “male.” The writing marks the mistake that involves a space destined for the entry and exit of bodies crossed by a signifier subject to drifts, erasures and mistakes around the signified. It is neither male nor female, but “male,” a signifier-other that borders on the real of what is already written with such incompleteness (Cf. Milner, 1978/2012).

Elza Soares has long sung that “the cheapest meat on the market is the black meat,” and rightly so, we know that in this world of human beings, we need to turn some people into “meat” because the condition of humanity before being a genetic, biological or natural data is the combination of historical determinations around ethnicity, race, class, gender and sexuality. The way the State calculates its “costs”—which weighs a lot or a little on the social balance about who is more or less of a citizen—proves how unequal the condition of human being is in this world (which is not one). When Butler (2009/2016) thinks about the unequal distribution of humanity, she wonders which frameworks shape certain lives as precarious and under what conditions it becomes difficult or even impossible. In this discussion, the body is immersed in the disciplinary and normalizing processes that shape recognition, but what is also at stake is what break the rules, which embarrasses the eye and escapes the classifications. From the discursive point of view, we also see in Pêcheux (1982/1990) an investment not only in the mechanism of the dominant ideology, but in the failures, the contradictions, in the sinuous movements of the senses (and of the subjects).

When Luísa Marilac, in her outburst, proposes: “You, woman, who is watching this right now, put yourself in my shoes, as if you were a transvestite,” she summons at the same time the limits of the identification processes, but also the relationship that is established between a place and a non-place. Bringing up the everyday mechanisms by which the restroom participates in the excluding functioning of cities, composing one of the non-places for trans subjects, is a practice of denunciation, among others, but it is also the establishment *of places of enunciation*, not just those in which the subject is (not) said, but also those in which they can say. When thinking about the relations between the places of enunciation and the discourse, Zoppi-Fontana (1999/2003) tensions the division of the right to enunciate and the effectiveness of this division in its effects of *legitimacy, truth, credibility, authorship, identification, and circulation*. This proposal is based on a theoretical affiliation to Discourse Analysis as proposed by Pêcheux and more specifically to the notion of *subject position* (Pêcheux, 1975/2009), considering the way in which the figure of ideological interpellation is central to this concept, but also in the way the subject of the discourse is thought through a contradictory relationship with such processes of interpellation. This allows us to problematize how historically subordinate and silent places emerge, interfering in stabilized directions. Thus, when Luísa Marilac and Maria Clara Spinelli, among many others, go public to expose their pains, their traumas, their “tics,” there is something beyond what is said about a given event: emerges a subject who asserts themselves in that place of subject. The event overflows: it is about saying who enters and who is kicked out, who can and who cannot, who looks and who is looked at.

This surplus in the event, which refers to the encounter between a memory and a current event (Pêcheux, 1983/2012), concerns to the fact that talking about the process of construction of meanings that constitute the laws of access to public restrooms for trans individuals is also touching on the many stabilized speeches that produce:

1. The erasure of trans subjects in the legislative discourse, which both by constrains and by calls for security, builds, on the one hand, a subject without affection, on the other, a sexual predator;
2. The naturalization of biological arguments, which, taken as the crude truth that precedes the discourse, work at the service of the cisgenerity imperative, taken as the sheer transparency of bodies and a dominant imaginary about masculinity (aggressive and protective) and femininity (vulnerable and incapable);
3. The political and theoretical reaction that runs through activist, institutional and intellectual production, but also daily practices through actions of repudiation, reports, complaints, texts, videos, and other material that come into dispute, claiming a dignified existence in the social environment.

The attempts, whether judicial or informal, to restrict the use of restrooms according to the users' gender identities or expressions should therefore be seen as ways of regulating and perpetuating the cisnormative binarism of gender. In this respect, the expulsion and embarrassment of transgender people in restrooms can be understood as a way of punishment for the transgression of gender norms (Bender-Baird, 2016, p. 987) and as such they must be combated to guarantee equity of rights desirable in a democratic and plural society that respects human dignity.

## Author Contributions

All authors listed have made a substantial, direct and intellectual contribution to the work, and approved it for publication.

## Conflict of Interest

The authors declare that the research was conducted in the absence of any commercial or financial relationships that could be construed as a potential conflict of interest.

## Footnotes

^1^https://www.youtube.com/watch?v=VJ_EuQHNm6E

^2^Maria Clara Spinelli is a theater, film, and television actress. She was the first transsexual actress to play a character of a cisgender woman on Rede Globo, in the soap opera A força do querer (2017).

^3^Expression by Lauretis (1989) to define the set of institutions and practical techniques and functions that (re)produce the truth of masculinity and femininity (Lauretis, 1989).

^4^On the one hand, we consider, from a discursive perspective, the constitutive relationship between the city and the subject (Orlandi, 2004), on the other hand, we also take into account the dialogues that such relations generate between Linguistics and History with respect to coercions and transgressions in the gender field (Cf. Chaves, 2015; Cestari, 2015, among other works developed in the Women in Discourse Research Group. https://www.iel.unicamp.br/br/content/mulheres-em-discurso).

^5^Historically, it is not difficult to think that this type of statement supports practices of violence that pervade several enunciative instances marked by unowned statements: “I don't know why I'm beating, but you know why you're being beaten,” “you were asking to be raped,” etc., in which an alleged knowledge of the victim participates in the network of arguments that support the aggressor's violence.

^6^As Preciado (2018) points out: “[…] Two opposing logics dominate the women's and men's restrooms. While the female bathroom is the reproduction of a domestic space in the middle of the public space, the male bathroom is an addendum to the public space, in which the laws of visibility and upright position are intensified, which traditionally defined the public space as a space of masculinity. While the women's bathroom operates as a mini-pan-optic, in which women collectively monitor their degree of heterosexual femininity and in which every sexual approach results in male aggression, the men's bathroom appears as a breeding ground for sexual experimentation.”

^7^https://www.select.art.br/lixo-e-genero-mijar-cagar-masculino-feminino/

^8^In this sense, it is also recurrent, in the speeches that discuss access to the women's bathroom for trans women, that the male enunciator takes a position occupying roles that express a family and/or personal relationship with women, such as the position of *father* and *husband*, saying that it is necessary to protect, for example, their daughters and wives from possible “sexual predators” in the bathrooms. We will also address this aspect later.

^9^We assume the definition of a transgender person as someone who does not identify themselves with the gender originally assigned to them at their birth, including, therefore, transsexual women and men. The considerations we make regarding the use of the female bathroom by transgender women in this article also apply, considering the Brazilian socio-cultural context, to transvestites, as this group also has a feminine gender identity and demands the use of the feminine bathroom, as well as transgender women. We use the expression “cisgender people” to designate people who are not transgender, and cisnormativity as the norm that establishes cisgenerity (the condition of cisgender people) as the desirable social standard.

^10^The arguments that supported racial segregation during the Jim Crow laws in the United States are analogous to arguments in favor of trans-exclusionary positions, as they both harbor similar fears about the need to protect women and children from a group of people perceived as capable to corrupt public morals, health, and order (Pogofsky, 2017, p. 753).

^11^We designate “trans-exclusionary bathroom laws” those laws that aim to prohibit the use of restrooms by transgender people according to their gender identities, and “trans-inclusive bathroom laws” those laws that aim to guarantee access to restrooms for transgender people according to their gender identities.

^12^Senate Bill 1306, South Carolina General Assembly 121st Session, 2016, http://www.scstatehouse.gov/billsearch.php?billnumbers=1306&session=121&summary=B.

^13^It is relevant to consider that access to rectification of official documents for transgender people may vary according to the legislation of each country and that minors in general may have to face greater bureaucracy and stricter legal requirements than trans adult people.

^14^Misgender is the act of mistakenly assigning a gender that does not match someone's gender identity.

^15^Levi and Redman (2010, p. 136) understand that forcing a transgender person to use a bathroom that is inconsistent with their gender identity is a form of discrimination, as it is based on disregarding the gender identity of transgender people while respecting the identity of cisgender people. Discrimination against transgender people leads to violence, poverty, and social isolation, in addition to negatively interfering with access to social, economic, and health support systems (Barnett et al., 2018, p. 237).

^16^We share the position of França et al. (2019), in understanding that one of the functions of the designation “gender ideology” is the negation of the ideological within the ideological.

^17^In the field of gender and American anti-Islam policies, we have the work of Judith Butler on the precariousness of life and the condition of ineluctable (Butler, 2009/2016). In the field of racial issues, Achille Mbembe's work on the construction of the racial subject as the hostile other (Mbembe, 2013/2018).

^18^On the other hand, other arguments against the access of trans women to women's restrooms are not justified by the appeal for safety, but rather by the basic belief that trans women are men and therefore could not use the women's bathroom (Wilson, 2016, p. 1400). A survey by Rudin et al. (2014) revealed that a significant number of participants understand that access to bathrooms for transgender people according to gender identity must be conditioned to the performance of sexual reassignment surgery, which implies the naturalization of public policies of invasive disciplinatory action of the corporeity of the population.

^19^The assumption that women's privacy in women's restrooms is guaranteed by the exclusion of men or people assigned with the male gender at birth is based on the assumption that only men or people assigned with the male gender at birth would be interested in invading that privacy (Levi and Redman, 2010, p. 163).

^20^Levi and Redman (2010, p. 154) point out that the laws that prohibited the use of clothes of a certain gender if someone had been assigned the other gender in the 1950s until the 1980s in the USA used the argument that they would prevent “fraud” and, with that, the violent attacks in the bathrooms. It is interesting to consider the materiality of the language in the constitution of such arguments, since the designation “gender fraud” puts at stake a relationship between “truth × lie,” “cunning × righteousness,” but where would be the gross truth of the gender in a state of purity and susceptible to verification?

^21^The authors (Barnett et al., 2018) were able to locate only one registered case of a transgender woman who committed a sexual offense in a women's bathroom (took pictures of the users of the bathroom without their permission).

^22^It is worth mentioning that in the context of the use of the women's bathroom, transgender women are often imagined as having “male anatomies” (Schilt and Westbrook, 2015, p. 29).

^23^Luisa Marilac is a youtuber, communicator, writer, and LGBTQ activist. In 2019 she published the biography “Eu, travesti: memórias de Luisa Marilac,” by Record publisher.

^24^https://www.youtube.com/watch?v=ARHLKPJPc7o&t=2

^25^https://www.youtube.com/watch?v=Bsrq8qv8Uig
